# Transcriptional repression of *p27* is essential for murine embryonic development

**DOI:** 10.1038/srep26244

**Published:** 2016-05-19

**Authors:** Youichi Teratake, Chisa Kuga, Yuta Hasegawa, Yoshiharu Sato, Masayasu Kitahashi, Lisa Fujimura, Haruko Watanabe-Takano, Akemi Sakamoto, Masafumi Arima, Takeshi Tokuhisa, Masahiko Hatano

**Affiliations:** 1 Department of Biomedical Science, Graduate School of Medicine, Chiba University, 1-8-1 Inohana Chuo-ku, Chiba city, Chiba, Japan; 2 Developmental Genetics, Graduate School of Medicine, Chiba University, 1-8-1 Inohana Chuo-ku, Chiba city, Chiba, Japan; 3 Biomedical Research Center, Chiba University, 1-8-1 Inohana Chuo-ku, Chiba city, Chiba, Japan.

## Abstract

The *Nczf* gene has been identified as one of Ncx target genes and encodes a novel KRAB zinc-finger protein, which functions as a sequence specific transcriptional repressor. In order to elucidate Nczf functions, we generated Nczf knockout (Nczf−/−) mice. Nczf−/− mice died around embryonic day 8.5 (E8.5) with small body size and impairment of axial rotation. Histopathological analysis revealed that the cell number decreased and pyknotic cells were occasionally observed. We examined the expression of cell cycle related genes in Nczf−/− mice. *p27* expression was increased in E8.0 Nczf−/− mice compared to that of wild type mice. Nczf knockdown by siRNA resulted in increased expression of *p27* in mouse embryonic fibroblasts (MEFs). Furthermore, *p27* promoter luciferase reporter gene analysis confirmed the regulation of *p27* mRNA expression by Nczf. Nczf−/−; p27−/− double knockout mice survived until E11.5 and the defect of axial rotation was restored. These data suggest that *p27* repression by Nczf is essential in the developing embryo.

Cell proliferation and differentiation are coordinated during development. Chromosome status is monitored at the G1 and G2 cell cycle checkpoints to start DNA replication and cell division, respectively. Many factors that regulate cell cycle entry, arrest, or progression have been identified. Cyclins and cyclin-dependent kinases (CDKs) are key players of cell cycle regulation^1^,^2^. CDKs form complex with their cyclin partner. Cyclin/CDK complex regulates progression of the cell cycle by phosphorylating their target substrates. Periodic oscillation of cyclins is a central event in cell cycle regulation. Cyclin/CDK complex activity is further regulated by positive and negative regulators. CDK activating kinase (CAK) is a multi-subunit protein complex that activates CDK/cyclin complex. On the other hand, CDK inhibitors (CDKIs) bind CDKs and negatively regulate CDK/cyclin activity. CDKIs are classified in two major categories, the INK4 family and the Cip/Kip family. The INK4 family proteins include p16^INK4A^, p15^INK4B^, p18^INK4C^, and p19^INK4D^. They specifically bind CDK4 and CDK6 and subsequently inhibit cyclin D binding. The Cip/Kip family includes p21^Cip1/Waf1^, p27^Kip1^, and p57^Kip2^. They inhibit CDK/cyclin activity, which is essential for G1 to S transition, and stop cell cycle progression into S phase. Gene targeting studies of these cell cycle regulators in mice revealed that they are largely dispensable for division of most embryonic and adult cells. So far, embryonic lethality at an early stage of embryogenesis was reported for Cdk1, cyclinB1 and cyclinA2 deficient mice. Some cell cycle regulator members are essential for only specific cell types and some are functionally redundant^3^. These studies also suggest the plasticity of mammalian embryonic cell cycle regulation and many important aspects of *in vivo* regulation of the cell cycle remain undiscovered.

*Nczf* (Ncx regulated zinc finger) was identified as a target gene of Ncx^4^. It contains an N-terminal Krüppel-associated box (Krab) domain and 11 Krüppel C2H2 type zinc finger domain at the C terminus. Krab zinc finger proteins constitute the largest family of transcriptional regulators encoded by higher vertebrates. They form a family of more than 400 active members in the human genome^5^. The Krab domain confers a potent transcriptional repressor function by specific interaction with a corepressor protein, KAP1 that recruits the chromatin deacetylation machinery such as histone deacetylase (HDAC)^6^,^7^.

Although most Krab zinc finger proteins function as transcriptional repressors, their respective target genes, underlying mechanisms, and physiological functions remain largely unknown. Recently, a Krab zinc finger protein, zinc finger, and BRCA1 interacting protein with Krab domain 1 (ZBRK1) was reported to repress the transcription of DNA damage responsible genes such as *gadd45a* and *p21*^8^,^9^,^10^. ZFP809 also belongs to the Krab zinc finger protein family. It specifically recognizes mammalian retroviruses and retroelements and silences their transcription together with KAP1 protein in embryonic stem cells^11^,^12^.

*Nczf* mRNA is ubiquitously expressed in adult and embryonic mouse tissues, whereas Ncx is specifically expressed in neural crest derived tissues^13^, suggesting that Nczf may exhibit a variety of biological functions in various situations. We previously demonstrated that Nczf expression is induced by various apoptosis inducing stimuli such as X-ray irradiation, dexamethasone, H_2_O_2_, and ultraviolet in mouse thymocytes or NIH3T3 cells^14^. However, its physiological function remains elusive.

To elucidate the role of Nczf in development, we disrupted *Nczf* by homologous recombination in ES cells and generated *Nczf* deficient (−/−) mice. Nczf−/− mice were embryonic lethal and cell proliferation was impaired. Molecular analysis revealed that *p27* mRNA expression was up-regulated in E8.5 Nczf−/− mice. Furthermore, Nczf knockdown in MEFs induced p27 expression and p27 promoter reporter gene analysis revealed that Nczf negatively regulates *p27* expression. Simultaneous deletion of *p27* could partially rescue the embryonic development defect caused by Nczf deficiency and prolonged the survival period. However, it could not rescue embryonic lethality, suggesting that Nczf regulates multiple target genes. The role of Nczf in development, cell proliferation, and survival is discussed.

## Results

### Generation of Nczf−/− mice

**Figure 1:**
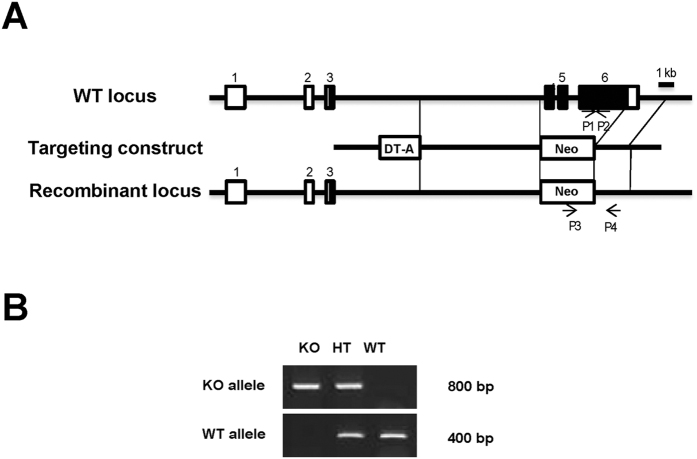
Targeted disruption of the *Nczf* gene. (**A**) The *Nczf* locus containing 6 exons and a portion of the 5′ and 3′ flanking regions is shown at the top. The targeting vector was designed to replace a DNA segment through exon 4 to exon 6 by a neomycin resistant gene cassette (NEO). A diphtheria toxin A gene cassette (DT-A) was used for a negative selection. The recombinant allele, resulting from homologous recombination, is depicted in the bottom panel. The coding exons or portions of exons are depicted by filled boxes and the open boxes denote the non-coding portions. The positions of the primers (P1 and P2) used for genotyping by PCR analysis are also indicated. The orientation of both NEO and DT-A was the same as that of *Nczf*. (**B**) PCR analysis of genomic DNA extracted from E8.5 mice. A primer set (P1 and P2) was used to detect the wild type (WT) allele (400 bp) and the primer set (P3 and P4) was used to detect *Nczf* knockout (KO) allele (800 bp). Whole image of the gel electrophoresis data is shown in supplementary information.

In order to examine the physiological role of Nczf in development, we disrupted *Nczf* in mice. *Nczf* exons 4 to 6, including the Krab zinc finger, were replaced by the neomycin resistant gene in embryonic stem cells (Fig. 1A). Correct targeting was confirmed by PCR genotyping (Fig. 1B). Heterozygous mutant mice (Nczf+/−) were fertile and showed no abnormality up to 2 years of age. The confirmed heterozygous mice were intercrossed to obtain homozygous (Nczf−/−) mice. No Nczf−/− mice were born alive among the 184 offspring from different heterozygous intercrosses. The ratio of heterozygous to wild-type mice was 2 to 1, indicating that *Nczf* loss was embryonic lethal. To determine the stage at which Nczf−/− embryos died during embryonic development, embryos were isolated from timed heterozygous intercrosses from embryonic day 14.5 (E14.5) to as early as E6.5. No viable Nczf−/− embryos were isolated at E10.5 or later. However, we were able to isolate small embryos at E9.5 and E8.5, which were subsequently confirmed to be Nczf−/− by PCR genotyping. Nczf−/− embryos at E9.5 and E8.5 exhibited a disrupted development compared with wild type and heterozygous littermates. Analysis of 75 embryos at E9.5 and 85 at E8.5 revealed that less Nczf−/− mice were obtained than expected based on the Mendelian frequency (Table 1: approximately 6.6% and 4.7% of embryos examined were Nczf−/− at E9.5 and E8.5, respectively). At E6.5, 19% of the examined embryos were homozygous mutants. We also collected blastocysts (E3.5) and determined that about 25% were Nczf−/− by PCR-genotyping. Taken together, these data indicate that *Nczf* disruption causes embryonic lethality and most of the Nczf−/− mice died by E9.5.

### Nczf disruption results in decreased cell proliferation and accelerated apoptosis

**Figure 2:**
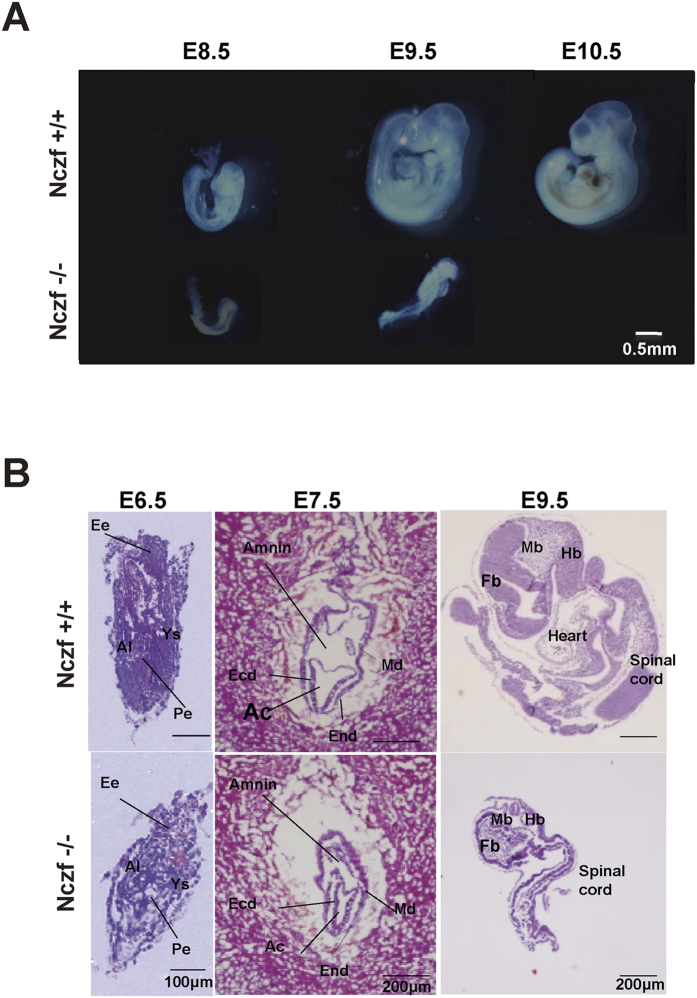
Morphological analysis of Nczf−/− mice. (**A**) Whole mount images of typical examples of Nczf−/− and their littermate embryos taken at E8.5, E9.5, and E10.5. (**B**) H&E staining section of Nczf−/− and their littermate embryos taken at E6.5 E7.5, and E9.5. Abbreviations; Al: Allantois, Ys: Yolk sac, Pe: Primitive ectoderm, Ee: Extra embryonic ectoderm, Md: Mesoderm, End: Endoderm, Ecd: Ectoderm, Ac: Amniotic cavity, Mb: Midbrain, Hb: Hindbrain, Fb: Forebrain.

To assess the effect of *Nczf* disruption in embryogenesis, we carried out macroscopic and histological examinations of the embryos. The process of “turning” is initiated in wild type E8.5 embryos and the process is normally completed by E9.0. E8.5 Nczf−/− embryos were small and remained unturned. At E9.5, the growth of Nczf−/− embryos was severely retarded. The embryos were smaller, unturned, and started to be resorbed (Fig. 2A). Histological analysis revealed that Nczf−/− embryos at E6.5 and E7.5 were smaller than wild type mice (Fig. 2B). Since no particular structural abnormality was detected at E6.5 and E7.5, Nczf−/− embryos underwent the gastrulation process normally and were transformed into multi-layered, three-chambered conceptus containing the mesoderm. Furthermore, trophoblast giant cells were observed in Nczf−/− extraembryonic tissues. At E9.5, neural plate cell layers were disorganized and a general hypocellularity became distinct in Nczf−/− embryos (Fig. 2B), suggesting a potential defect in cell proliferation. In addition, cells with condensed nuclei were occasionally observed in E8.5 Nczf−/− embryos. Thus, failure in cellular proliferation is likely the major cause of embryonic lethality at E9.5 in Nczf−/− mice.

### Deregulation of *p27* expression in Nczf−/− embryos

**Figure 3:**
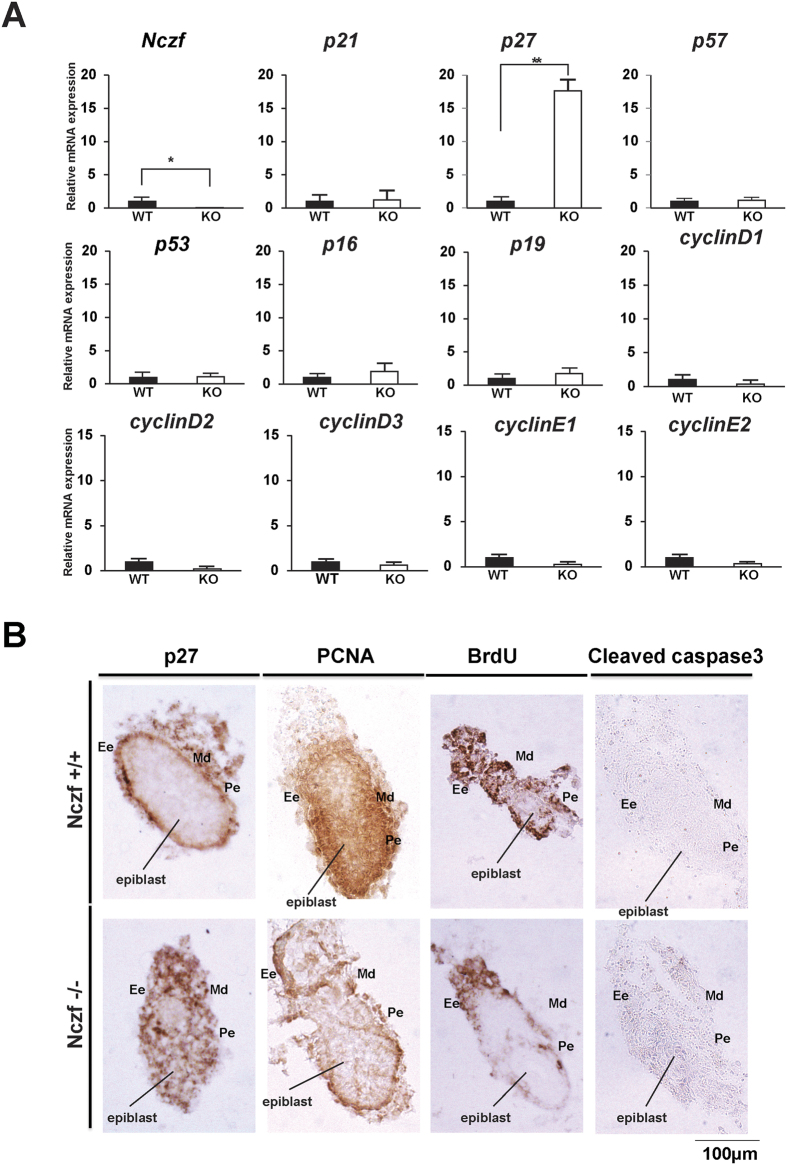
Expression of CDK inhibitor and cyclin mRNAs in E8.0 Nczf−/− mice and immunohistochemical analysis at E6.5. (**A**) *Nczf, p53, p19, p21, p27, p57, p16,* cyclin D1, cyclin D2, cyclin D3, cyclin E1, and cyclin E2 mRNA expression in wild type (closed bars) and Nczf−/− mice (open bars) at E8.0. Target-specific mRNA levels were assessed by real time PCR and normalized to *Gapdh* mRNA (n = 4–5). The expression in wild type mice was set to 1 and the relative amount of mRNA expressed in Nczf−/− mice was calculated. *P < 0.01. (**B**) Whole images of E6.5 embryos from wild type and Nczf−/− mice immunostained with p27, PCNA, BrdU and active caspase3 antibodies. Scale bar: 100 μm. Abbreviations; Pe: Primitive ectoderm, Ee: Extra embryonic ectoderm, Md: Mesoderm.

To investigate the molecular link between Nczf deficiency and cell proliferation, we examined the expression of cell cycle regulator genes in Nczf−/− embryos. Among the potential candidate genes, expression of the CDK inhibitor, *p27* gene, was markedly up-regulated in the E8.5 Nczf−/− embryos, while its expression was barely detectable in wild type embryos. No significant difference was found in other cell cycle regulating genes, including *p21, p57, p53, p16, p19,* cyclin D, and cyclin E (Fig. 3A). Histological analysis revealed that cell proliferation was impaired and p27 expression was up-regulated as early as E6.5 (Fig. 3B). PCNA positive cells were observed throughout epiblast in wild type mouse, whereas no PCNA positive cells were observed in the epiblast of the Nczf−/− mouse. These were also confirmed by BrdU labeling experiment. After 60 min of BrdU administration, incorporated epiblast and extraembryonic ectoderm cells were observed in wild type mouse. In contrast, there were no BrdU incorporated epiblast cells in Nczf−/− mouse. Expression site of p27 was inversely correlated with that of PCNA. Many p27 positive cells were observed in the embryonic ectoderm of Nczf−/− mouse, whereas no p27 positive cell was detected in wild type mouse. On the other hand, there was no difference in the expression of active caspase3 and no sign of apoptosis was observed at this stage (Fig. 3B). Thus, *Nczf* loss resulted in a deregulated expression of p27, impairment of cell proliferation, and developmental arrest from early embryonic stages.

### *p27* is a molecular target of Nczf

**Figure 4:**
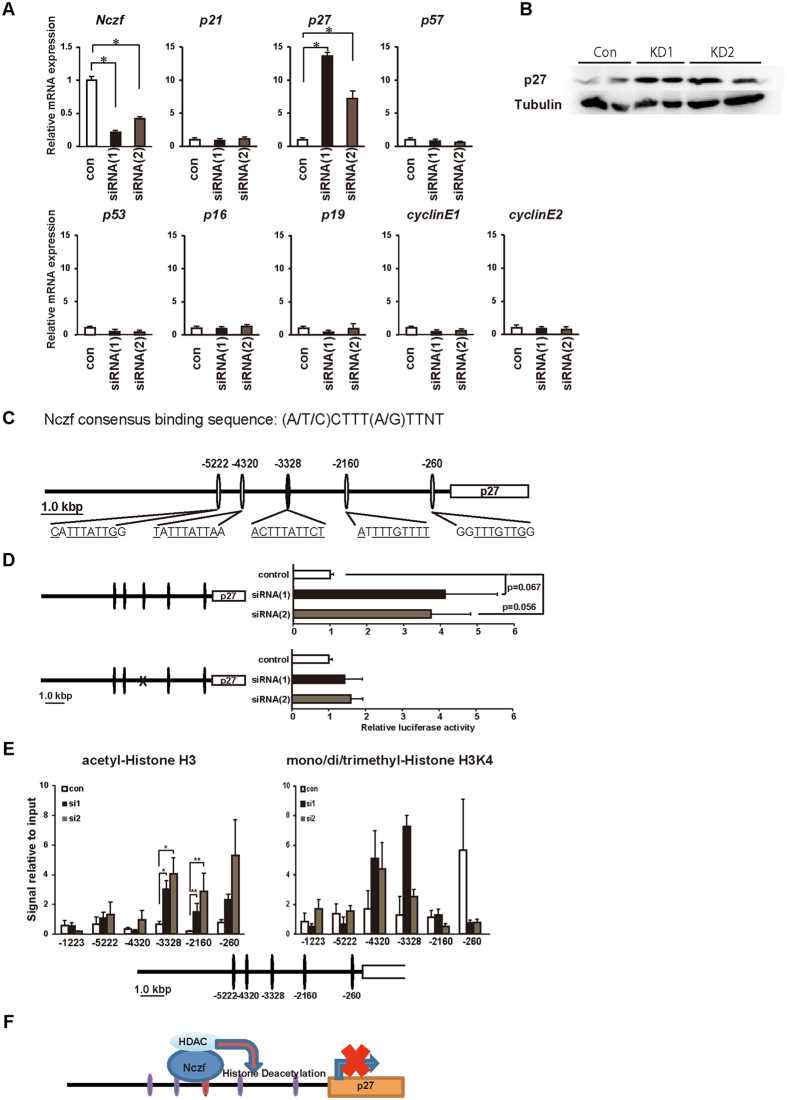
Regulation of *p27* mRNA expression by Nczf. (**A**) *Nczf, p53, p21, p27, p57, p16, p19, cyclin D1, cyclin D2, cyclin D3, cyclin E1, and cyclin E2* mRNA expression in Nczf knockdown MEFs. MEFs were transfected with control or two independent siRNAs. Target-specific mRNA levels were assessed by real time PCR and normalized to *Gapdh* mRNA (n = 5–8). The expression in wild type was set to 1 and the relative expression of the genes of interest in Nczf−/− MEFs was calculated. (**B**) Expression of p27 protein in MEFs after Nczf knock down. MEFs from two independent experiments in each siRNA transfection were examined. Tubulin is shown as a loading control. Whole image of the blot is shown in supplementary information. (**C**) A schematic representation of Nczf binding sequence in a 5′ flanking region of *p27* gene. Nucleotides that match the consensus sequence were underlined. There are multiple potential Nczf binding sites, one of them is 100% identical to the Nczf consensus binding motif (−3328), four of them are 70% compatible with Nczf consensus binding motif (−5222, −4320, −2160, and −260). (**D**) (left panel): A schematic representation of a 5.5 kb 5′ flanking region upstream of the translation start site of the *p27* gene and reporter gene constructs. (Right panel): p27 luciferase reporter assay. MEFs were transfected with intact (p27-Luc) or Nczf binding site mutated p27 reporter constructs (p27(del)-Luc) along with Nczf knockdown vector. The cells were subjected to firefly-luciferase reporter assays with Renilla luciferase. All data of the luciferase reporter assay above are means of three independent experiments (n = 7). (**E**) Binding of acetyl-Histone H3 and mono/di/trimethyl-Histone H3K4 to the p27 promoter region after Nczf knockdown by 2 independent siRNAs (siRNA1 and siRNA2: closed and shaded bars, respectively) were analyzed by ChIP assay. Quantitative PCR was performed on chromatin fragments isolated before and after immunoprecipitation using anti-acetyl-Histone H3 and anti-mono/di/trimethyl-histone H3K4. Numbers in the schematic representation show targeted regions. The region −1223 fragment was used for control. (**F**) A schematic summary of Nczf mediated transcriptional repression of *p27* gene. Abbreviations; HDAC: histone deacetylase.

Since Nczf functions as a transcriptional repressor, we further examined the regulation of *p27* mRNA expression by Nczf. We knocked down *Nczf* using siRNA in MEFs and the mRNA expression of the above examined cell cycle regulator genes was determined by qRT-PCR (Fig. 4A). In agreement with previous data, *p27* mRNA expression was de-repressed by Nczf knockdown in MEFs. Increased expression of p27 protein was also confirmed by western blotting (Fig. 4B). The expression of other cell cycle related genes such as *p21, p57, p16, p19, cyclin Ds,* and *cyclin Es* was not affected by Nczf knockdown. Next, we searched for the Nczf binding consensus sequences in the murine *p27* promoter region and identified 5 potential Nczf binding motifs within 5 kb from the translational start site of the *p27* gene (Fig. 4C). One of them, located 3328 bp upstream of the translational start site was 100% identical to the consensus motif and the others were about 70% identical. Reporter gene assay using a p27 luciferase construct and Nczf siRNA knockdown plasmids was performed in MEFs. Luciferase activity of the reporter gene linked to the *p27* promoter region was increased when Nczf was knocked down by 2 different siRNAs (Fig. 4D: upper figure). In contrast, when the 100% matched consensus motif was deleted in the reporter construct, no increase of luciferase activity was observed after Nczf knockdown in MEFs (Fig. 4D: lower figure). To examine the chromatin status at the *p27* genomic locus, chromatin immunoprecipitation (ChIP) assay was performed using Nczf knockdown MEFs. Acetylation of histone H3 and mono- and tri-methylation of H3K4 are markers of transcriptionally active chromatin. Histone H3 acetylation at position −3328, −2160, and −260 was increased in the *p27* genomic locus after Nczf knockdown. H3K4 mono/di/trimethylation at position −4320 and −3328 was also enhanced after Nczf knockdown (Fig. 4E). These results suggest that Nczf represses *p27* mRNA expression by modifying chromatin structure.

### Partial rescue of Nczf−/− embryos by simultaneous deletion of p27

**Figure 5:**
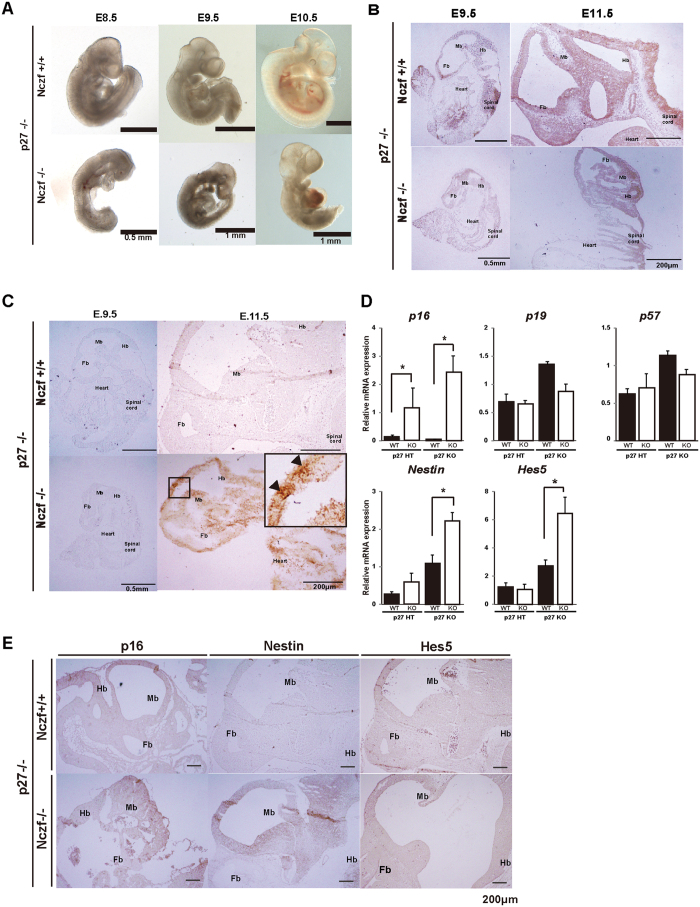
Partial rescue of Nczf−/− phenotype by simultaneous deletion of *p27*. (**A**) Whole mount images of typical examples of Nczf−/−; p27−/− littermate embryos taken at E8.5, E9.5, and E10.5. Scale bars 500 μm (E.8.5) and 1.0 mm (E9.5 and E10.5) (**B**) PCNA immunostaining of E9.5 and E11.5 embryos of Nczf+/+; p27−/− and Nczf−/−; p27−/− mice. (**C**) Cleaved caspase3 immunostaining of E9.5 and E11.5 Nczf+/+; p27−/− and Nczf−/−; p27−/− embryos. High magnification of boxed area is shown in inset. Arrows in the inset indicate cleaved caspase 3 positive cells in mesencephalon. Scale bars in (**B,C**) 500 μm (E.9.5) and 200 μm (E11.5). (**D**) *p57, p16, p19,* nestin and *Hes5* mRNA expression in E10.5 Nczf+/+ and Nczf−/− embryos under p27−/− conditions. Target-specific mRNA levels were assessed by real time PCR and normalized to *Gapdh* mRNA (n = 3–5). (**E**) p16, Nestin and Hes5 immunostaining of E11.5 embryos of Nczf+/+; p27−/− and Nczf−/−; p27−/− mice. Scale bars: 200 μm. Abbreviations; Mb: Midbrain, Hb: Hindbrain, Fb: Forebrain.

Since *p27* mRNA expression is elevated in E8.5 Nczf−/− embryos and the elevation may cause the impairment of cell proliferation and embryonic lethality, we generated Nczf−/−; p27−/− double knockout mice to examine the effect of p27 in the Nczf−/− genotype. Mice heterozygous for both Nczf and p27 (Nczf+/−; p27+/−) were intercrossed and genotyping was performed at weaning. No homozygous mutants for both Nczf and p27 were obtained, indicating that *p27* deletion alone cannot fully rescue the Nczf−/− embryonic lethality (Table 2). To examine whether p27 deletion could partially rescue the Nczf−/− phenotype, embryos were isolated from timed double heterozygous (Nczf+/−; p27+/−) intercrosses. No viable Nczf−/− embryos were obtained at E12.5 or thereafter. However, we were able to identify Nczf−/−; p27−/− double knockout mice at E11.5 (Table 2). Furthermore, we could identify the Nczf−/−; p27+/− mice at E11.5, E10.5, and E9.5 although the number was less than expected based on the Mendelian frequency. E10.5 Nczf−/−; p27−/− double knockout (DKO) mice were smaller, but some of them underwent “turning” of the body (Fig. 5A). Immunohistochemistry revealed that cell proliferation was partially restored in DKO embryos (Fig. 5B). At E11.5, PCNA-positive cells were observed in the forebrain, midbrain, and hindbrain areas. However, cleaved caspase positive cells also increased in the forebrain and midbrain areas, suggesting that cell proliferation and cell death occurred at this stage (Fig. 5C). To examine the status of cell cycle regulator genes, mRNA was isolated from Nczf−/−; p27−/−, Nczf+/+; p27−/−, Nczf−/−; p27+/− and Nczf+/+; p27+/− embryos at E10.5 and qRT-PCR was performed (Fig. 5D). *p16* mRNA expression was strongly up-regulated in Nczf−/− embryos in *p27* null and heterozygous conditions. We further analyzed mRNA expression by RNA microarray. Several genes related to neuronal differentiation were up-regulated in Nczf−/−; p27−/− mice compared to Nczf+/−; p27−/− mice. Nestin, which is expressed in neuronal progenitor cells and during early neurogenesis, was up-regulated in DKO mice. *Hes5,* which encodes a helix-loop-helix repressor protein important for neurogenesis, was also up-regulated in DKO mice. Furthermore, the elevated expression of these genes was confirmed in protein level by immunohistochemistry. Expression of p16, Nestin and Hes5 are elevated in midbrain and forebrain areas in Nczf−/−; p27−/− mice (Fig. 5E). These data indicate that Nczf not only negatively regulates *p27* mRNA expression, but also functions through multiple regulators to support cell proliferation, differentiation, and survival during embryogenesis.

## Discussion

In this study, we disrupted Nczf, a Krab zinc finger coding protein, in mice and showed that Nczf plays an important role in cell proliferation and embryonic development. Our data demonstrated that Nczf deficient embryos became disorganized at E8.5, severely growth-retarded, and eventually died by E9.5. No particular structure was missing and overall hypocellularity was observed at this stage in Nczf−/− embryos, suggesting that the abnormality was mainly due to a defect in cell proliferation rather than during differentiation.

The most significant and complicated changes in the murine embryo occur between E8.5 and E9.5, when organogenesis is greatly accelerated and the process of “turning” is completed simultaneously. Continuous and rapid cell proliferation is required for the expansion of progenitor cells to establish the body plan within a limited time of embryogenesis.

A cyclin dependent kinase inhibitor, p27, inhibits cell cycle progression at the G1 phase^15^,^16^. p27 expression is normally not detectable from E6.5 until E13.5–14.5^16^. Intracellular abundance of p27 is regulated by several different mechanisms. Regulation of p27 expression mainly depends on proteasome-mediated degradation, Skp2-SCF-mediated ubiquitination, and cyclin E-Cdk2-mediated phosphorylation of p27Thr187^18^,^19^,^20^,^21^,^22^. In addition to these post-translational regulation mechanisms, transcriptional- or miRNA-mediated regulation was also reported. STAT1 and FOXO3a activates p27 transcription. Sirtuin1 and Sirtuin2 deacetylate FOXO3a protein and increase FOXO3a DNA binding activity, resulting in elevated expression of its target gene, p27^kip1 ^22. On the other hand, c-myc, AP-1, and E2F1 negatively regulates its expression^24^,^25^,^26^,^27^. In cancer cells, miR-221 and miR-222 repress p27 expression^28^,^29^,^30^,^31^. Our data demonstrated that Nczf negatively regulates p27 mRNA expression at the transcriptional level. Thus, p27 de-repression by Nczf disruption likely resulted in cell proliferation failure, leading to embryonic lethality at E9.5. Inhibition of p27 degradation pathways also causes accumulation of p27 protein. Genetic disruption of the component of SCF E3 ubiquitin ligases such as RBX or Jab1 resulted in p27 accumulation and early embryonic lethality^32^,^33^,^34^,^35^,^36^. These deficient mice died at E6.5, three days earlier than Nczf deficient mice, due to failure in cell proliferation during gastrulation. These facts suggest that ubiquitin mediated protein degradation is the major pathway for the regulation of cellular amount of p27 during early embryogenesis. This may explain the relatively longer survival of Nczf−/− mice. It is possible that the intact protein degradation machinery in *Nczf* deficient mice could compensate the up-regulated *p27* transcription until E7.5. After E8.5, a dramatic change in shape and size occurs in embryos and *p27* expression should be actively shut off at the transcriptional level. De-repression of *p27* causes cell cycle arrest, which induces apoptosis and developmental arrest at this stage.

Previous reports demonstrated that p27−/− mice are viable with about 30% increased body weight^37^,^38^. Thus, we hypothesized that, if *p27* de-repression is solely responsible for proliferation failure and embryonic lethality in Nczf−/− embryos, simultaneous deletion of *p27* and *Nczf* could rescue the phenotype. Interestingly, not only homozygous but also heterozygous deletion of *p27* could rescue the Nczf−/− phenotype until E11.5, suggesting that the amount of p27 is critical at this developmental stage. Failure to rescue the Nczf−/− embryonic lethality by simultaneous deletion of *p27* and *Nczf* indicates that lethality induced by *Nczf* disruption is likely due to a multifactorial mechanism and Nczf functions through multiple regulators to support cell survival, proliferation, and differentiation during embryogenesis. *p16* expression was significantly up-regulated in Nczf−/−; p27−/− double knockout embryos at E10.5. p16 is a member of the cyclin-dependent kinase 4 inhibitors that binds to and inhibits the activities of CDK4 and CDK6 at the G1 cell cycle checkpoint and is a major inducer of senescence^38^. We identified potential Nczf binding motif sequences in the regulatory region of the *p16* gene. Thus, Nczf may directly repress *p16* expression in later embryonic stages. p16 expression is also induced in response to various environmental and cellular stresses^40^,^41^. Another possibility is that under Nczf−/−; p27−/− conditions, embryonic cells continue to proliferate and escape from lethality at E9.5. However, environmental and cellular stresses caused by cell proliferation and differentiation accumulate, which leads to *p16* up-regulation and cellular senescence. Interestingly, MEFs isolated from E10.5 Nczf−/−; p27−/− embryos can survive more than 2 weeks *in vitro*, but they do not proliferate. Nczf not only regulates *p27* expression, but may also play a crucial role in the protection of cells from various stresses. Besides *p27* and *p16*, the expression of neuron specific genes such as nestin and *Hes5* is de-repressed in the double KO mice at E10.5. Nestin, an intermediate filament protein, is expressed in the neuroepithelium of the developing neural tube and is a marker for multipotent neural stem cells^41^. Hes5, a helix-loop-helix transcription factor, is known as a regulator of neural stem cell differentiation^42^. Several Nczf binding sequences are also identified in both nestin and *Hes5* genes. In E8.5, there were no differences in the expression of *Nestin* and *Hes5* between wild type and Nczf−/− mice (data not shown). Nczf may repress the expression of genes related to neural differentiation, which are unnecessary in later developmental stages.

Generally, Krab zinc finger proteins are considered sequence specific transcriptional repressors by recruiting HDAC to the Krab domain. Recent studies described that some members of the Krab zinc finger protein family are responsible for silencing the transcription of endogenous retroviruses. The Krab domain recruits KAP1 and H3K9 methyltransferase ESET, and the zinc finger DNA binding domain determines the specificity of genomic retroviral integration sites^12^,^44^. KAP1-deficient embryos died at E5.5 and the deficient mice or KO ES cells exhibited a marked up-regulation of endogenous retroviral elements^11^. We examined the expression of several endogenous retroviral elements in Nczf−/− embryos and Nczf−/− ES cells. No significant increase of the elements was detected in *Nczf* deficient conditions (data not shown).

In summary, this study revealed that Nczf is essential for embryonic development and cell proliferation by negatively regulating *p27* mRNA expression in mice. Our data also underscore the importance of the amount of *p27* in the regulation of rapid cell proliferation during embryogenesis.

## Methods

### Animals

Mice were purchased from Japan CLEA. P27 deficient mice were kindly provided by RIKEN Bioresource Center and Dr. K-I. Nakayama (Kyushu University)^36^. All mice used were housed on a 12 h day/night cycle under specific pathogen free conditions. All experimental procedures were approved by the Institutional Animal Care and Use Committees of Chiba University and were carried out in accordance with the National Institute of Health guidelines.

### Targeting disruption of the *Nczf* gene

A murine *Nczf* genomic clone was isolated from a 129/Sv genomic library. A targeting vector was constructed by replacement of a fragment including exons 3 to 6 by a neomycin resistance cassette. The diphtheria toxin A gene was inserted upstream of the long arm. The linearized targeting vector was transfected into R1 embryonic stem cells by electroporation. Homologous recombination in the G418 selected clones was screened by PCR. Two independent clones were used to generate chimeric mice by the aggregation method with slight modifications^44^. The heterozygous mutant mice were interbred to obtain homozygous mutant mice.

### Genotyping of mutant mice by PCR

For genotyping of *Nczf* or *p27* mutant mice, genomic DNA was isolated from ES cells or mice tail tips and were genotyped using the following primer pairs.

*Nczf* wild-type (WT) allele: 5′-AGAAGAGTCGAGAGAGACATTTGTGGCAAGTT-3′ and 5′-TTACTTTCAAGGCATTTACAAGATTTTC-3′;

*Nczf* KO allele: 5′-ACCGGACAGGTCGGTCTTGACAAAA-3′ and 5′-CTGGACTGTTTCACGCTTCC-3′.

*p27* WT allele: 5′-CCTGGAGCGGATGGACGCCAGACA-3′ and 5′-CACCAAATGCCGGTCCTCAGAGTT 3′;

*p27* KO allele: 5′-GGCTATTGGCTCAAAACGAACCTC-3′ and 5′-ATGCTCCAGACTGCCTTGGGAAAA-3′.

### Histological analysis

Animals were perfused with a solution of 4% paraformaldehyde (PFA) in 0.1 M phosphate buffer (pH 7.4). Embryos were isolated from mice and postfixed with 4% PFA for 12 h. The samples were paraffin embedded and sectioned at 5 μm thickness. Hematoxylin and eosin (H&E) staining was performed using a standard protocol.

### BrdU labelling of embryos

Labelling of proliferating cells with BrdU was performed as described^45^. Pregnant mice were injected intraperitoneally with BrdU (Roche, 280879) (2 mg per mouse) at E6.5 and sacrificed after 60 min.

### Immunohistochemistry

Embryos were fixed in 4% PFA overnight, embedded into OCT compound, and frozen. Frozen samples were sectioned at 5 μm thickness, dried up on slide glass, fixed in 4% PFA, and endogenous peroxidase activity was quenched by incubating the slides with 0.3% hydrogen peroxide. The sections were subjected to antigen retrieval by microwaving in sodium citrate (0.01 M, pH 6.0) as the antigen unmasking solution and then incubated overnight at 4 °C with the primary antibody. Antibodies used for this experiment are as follows: PCNA (PC 10, monoclonal, Sigma-Aldrich, St Louis, MO, USA), p27 (mouse monoclonal, Cell Signaling Technology, Danvers, MA, USA), activated caspase-3 (Cell Signaling Technology), BrdU (Bu20a, mouse monoclonal, Cell Signaling Technology), Nestin (clone 401, monoclonal, Merck Millipore), Hes5 (AB5708, polyclonal, Merck Millipore), and CDKN2A/p16INK4a (2D9A12, monoclonal, abcam).

Immunostaining with the mouse monoclonal antibody was carried out using an ABC kit (Vector Laboratories, Burlingame, CA, USA). After incubation with antibodies, sections were washed three times in PBS and then incubated with the appropriate biotinylated secondary antibody (DAKO and Vector Laboratories) followed by StreptABComplex/HRP (DAKO and Vector Laboratories) following the manufacturer’s instructions.

### Quantitative PCR (qPCR) analysis

Total RNA was extracted by RNeasy Mini Kit (QIAGEN, Hilden, Germany) or by the Trizol method and reverse transcribed with the SuperScript® VILO™ cDNA Synthesis Kit (Invitrogen, Carlsbad, CA, USA) according to the manufacturer’s protocol. qPCR with target specific primers was performed using Power SYBRGreen Master Mix (Applied Biosystems, Foster City, CA, USA). Conditions for each target were validated by standard and melting curve analyses. Target-specific amplification was normalized to a *Gapdh* control. Primer sets for each gene were as follows:

*Gapdh* 5′-TGTGTCCGTCGTGGATCTGA-3′ and 5′-TTGCTGTTGAAGTCGCAGGAG-3′,

*Nczf*: forward 5′-CAGAATCTCAACGACGCTCAGA-3′ and reverse 5′-AACTCAGGTTTAGGGATGCATTG-3′,

*p21*: forward 5′-CTGTCTTGCACTCTGGTGTCTGA-3′ and reverse 5′-CCAATCTGCGCTTGGAGTGA-3′

*p27*: forward 5′-CATTAACCCACCGGAGCTGTTTAC-3′ and reverse 5′-GGTTAGCGGAGCAGTGTCCA-3′

*p57*: forward 5′-CAGAATCTCAACGACGCTCAGA-3′ and reverse 5′-AACTCAGGTTTAGGGATGCATTG-3′

*p16*: forward 5′-TTTCGTGAACATGTTGTTGAGGCTA-3′ and reverse 5′-GCTACGTGAACGTTGCCCATC-3′

*p19*: forward 5′-CCTGAAGGTTCTGGTGGAGCA-3′ and reverse 5′-CTGTGGTGGAGATCAGATTCAGGA-3′

Cyclin D1: forward 5′-GCGTACCCTGACACCAATCTC-3′ and reverse 5′-ACTTGAAGTAAGATACGGAGGGC-3′

Cyclin D2: forward 5′-ACCTCCCGCAGTGTTCCTATT-3′ and reverse 5′-CACAGACCTCTAGCATCCAGG-3′

Cyclin D3: forward 5′-TGCGTGCAAAAGGAGATCAAG-3′ and reverse 5′-GGACAGGTAGCGATCCAGGT-3′

Cyclin E1: forward 5′-GACATAGACATTCAGCCAGGACACA-3′ and reverse 5′-TCCAAAGTTGCACCAGTTTGCTTA-3′

Cyclin E2: forward 5′-TCTGTGCATTCTAGCATCGACTC-3′ and reverse 5′-AAGGCACCATCGTCTACACATTC-3′

*Hes5*: forward 5′-AGTCCCAAGGAGAAAAACCGA-3′ and reverse: 5′-GCTGTGTTTCAGGTAGCTGAC-3′

Nestin: forward 5′-AGAGTCAGATCGCTCAGATCC-3′ and reverse: 5′- GCAGAGTCCTGTATGTAGCCAC-3′

### Cell culture

MEFs were isolated from E10.5–E13.5 embryos. The embryos were washed 3 times with PBS. The tissue was then minced with a scalpel and digested with 0.05% trypsin solution containing 0.53 mM EDTA (Invitrogen) for 30 min at 37 °C. Trypsin was inactivated by addition of a 15-fold excess of MEF medium. The cells from each embryo were placed into a 100-mm dish and cultured at 37 °C in a 5% CO_2_ incubator in DMEM (Invitrogen) supplemented with 10% FBS.

### Knockdown assay

Selected target sequences (control: CGTCAAACGAAGATGTGAA, siRNA1: GTATTGTAGAGCGATATAA, and siRNA2: TAGAGTGTTGATCAGAATA) were cloned in RNAi-Ready pSIREN-RetroQ-ZsGreen Vector (Clontech, Mountain View, CA, USA). These vectors were transfected into Plat-E cells as a packaging for producing retroviruses using Fugene (Roche, Basel, Switzerland). Transfected Plat-E cells were cultured at 37 °C in a 5% CO_2_ incubator in DMEM containing 10% FBS for 48 h. The virus-containing medium was then collected from packaging cells. MEFs were plated one day prior to the beginning of this procedure in 24-well plates. For retroviral infection, 0.5 mL of medium containing the virus and polybrene (8 μL/mL) were added per well and the cells were incubated 32 °C in a 5% CO_2_ incubator for 4 h. The medium was then replaced with fresh medium and the cells were incubated at 37 °C in a 5% CO_2_ incubator in DMEM containing 10% FBS.

### Western blot analysis

Cultured cells were washed by PBS three times and were lysed by SDS sampling buffer then heat 95–100 °C for 10 min. Samples with identical protein quantities were then separated by using 12% sodium dodecyl sulfate (SDS) polyacrylamide gel electrophoresis (PAGE), and transferred onto a PVDF membrane. The membrane was incubated at room temperature in blocking solution (5% skim milk,0.1% tween-20 in TBS) for 1 h, followed by a 2-h incubation in blocking solution containing an appropriate dilution of primary antibody (Anti-p27 [Cell Signaling Technology], anti-α tubulin [monoclonal, Wako]). After washing, the membrane was incubated in PBS containing goat anti-mouse IgG conjugated with horseradish peroxidase for 1 h. The membranes were washed and the positive signals developed with enhanced chemi-luminescence reagent (Luminate forte, Millipre).

### Luciferase reporter gene analysis

The BglII–HindIII fragment of mouse *p27*^*KIP1*^ extending from the 5′-UTR to the promoter region was subcloned into pGL3 basic vector (Promega, Madison, WI, USA) to construct p27-Luc vector. The primers used for PCR were as follows: *p27*: forward, 5′-CAAAACCGAACAAAAGCGAAACGCCA-3′, reverse 5′-GATACTCTCCCCTTCCTTTGCCTTGTC-3′. The P27(del)-Luc vector was produced from p27-Luc vector by PCR with primers 5′-GTGTTGTGGGCTGCCCGTGAG-3′ and 5′-CACAACACCCGACGGGCACTC-3′ using KOD-Plus-Mutagenesis kit (Toyobo, Osaka, Japan). All constructs were confirmed by sequencing.

For transfection and luciferase assay, MEFs were cultured in DMEM with 10% fetal bovine serum. On day 0, cells were plated on a 24-well plate at 2.5 × 10^4^ per well. On day 1, each luciferase reporter plasmid (0.25 μg) and pRL-SV40 reference plasmid (0.02 μg) (Promega) were transfected into cells using the transfection reagent Xfect (Takara, Tokyo, Japan) according to the manufacturer’s protocol. Four hours after transfection, cells were placed into fresh medium, followed by culture for 2 days before harvesting. The luciferase activity was measured and normalized to the activity of the co-transfected pRL-SV40 Renilla luciferase reporter.

### ChIP assay

ChIP assay was performed using a SimpleChIP Plus Enzymatic Chromatin IP kit (Cell Signaling Technology) following the manufacturer’s instructions. Briefly, MEFs infected with each siRNA expressing virus were cultured for 48 h and harvested. After cross-linking and chromatin digestion, antibodies against acetyl-Histone H3 (Polyclonal, MILLIPORE) and mono/di/trimethyl-histone H3(lys4)(Monoclonal, clone AW304, MILLIPORE) were used for chromatin immunoprecipitation.

Quantitative PCR to amplify the immunoprecipitated DNA fragments of *p27* regulatory region was performed using step one plus (Applied Biosystems) with the primers listed below.

Primer-260; forward: 5′-GACCACCTTCCCTCCTGATATATGC-3′, reverse: 5′-TACACAGAGAAACCCTGTCTCGGAA-3′,

Primer-2160; forward: 5′-ACTGTTTATTCTTCTGTCCCTCCC-3′, reverse: 5′-CCACATAGCTTGTTGTTTGCTGCCCTT-3′,

Primer-3328; forward: 5′-AGCAAACTTTGATGAAGGGCAGTG-3′, reverse: 5′-AGAGCACAGTCCCTTGTAGAAAGT-3′,

Primer-4320; forward: 5′-CAAGACAACCTGAACACAGTGGG-3′, reverse: 5′-GGATGCTCAGCAAGACCAAGAGGA-3′,

Primer-5222; forward: 5′-TCTCCGGCCGTTTGGCTAGTTTG-3′, reverse: 5′-GACATTGGCTGGTCGCGTGACTA-3′,

Primer-1223 (control); forward: 5′-CAATGCCATGCTCTCTCTTCTCTG-3′, reverse: 5′-CCCGTTTAAGAACACTTCTTCAGC-3′.

The signal relative to input was calculated as described by the manufacturer.

### Microarray analysis

RNA from E10.5 Nczf−/−; p27−/+ and Nczf+/−; p27−/+ MEFs was isolated and subjected to microarray analysis. Expression profiles were analyzed by SurePrint G3 Mouse GE 8 × 60K Microarray (Agilent, Santa Clara, CA, USA).

### Statistical analysis

The data are presented as the mean ± standard error of the mean (SEM). Differences in mean values between groups were analyzed using one-way ANOVA followed by pairwise comparisons using the Bonferroni post hoc test. P values < 0.05 were defined as significant.

## Additional Information

**How to cite this article**: Teratake, Y. *et al*. Transcriptional repression of *p27* is essential for murine embryonic development. *Sci. Rep.* **6**, 26244; doi: 10.1038/srep26244 (2016).

## Supplementary Material

Supplementary Information

## Figures and Tables

**Table 1:** Genotypes of Nczf mutant mice.

	**WT**	**HT**	**KO**	**Total**
3wks	63	121	0	184
E14.5	26	50	0	76
E10.5	14	28	0	42
E9.5	22	48	5*	75
E8.5	26	55	4	85
E6.5	23	45	16	84

Mice or embryos from Nczf heterozygous intercrosses were genotyped by PCR.

*Some E9.5 Nczf KO embryos were almost absorbed.

**Table 2:** Genotypes of Nczf mutant mice or embryos in the *p27* null background.

**p27 status Nczf status**	**WT**			**HT**			**KO**			**Total**
	**WT**	**HT**	**KO**	**WT**	**HT**	**KO**	**WT**	**HT**	**KO**	
3wks	12	15	0	20	46	0	12	21	0	126
E12.5	11	19	0	13	17	0	10	14	0	84
E11.5	5	10	0	5	7	2	3	5	1	38
E10.5	9	16	0	8	17	5	10	19	7	91
E9.5	10	15	0	16	20	6	11	16	7	101

Mice or embryos from Nczf+/−; p27+/− intercrosses were genotyped by PCR.
