# Supplementary material for: Transcriptional repression of p27 is essential for murine embryonic development
Source: Sci Rep. 2016 May 19;6:26244. doi: 10.1038/srep26244 (PMC4872541; doi:10.1038/srep26244)
Supplement: Supplementary Information [file srep26244-s1.pdf]

Supplementary information

Title:

Transcriptional repression of *p27* is essential for murine embryonic development

Youichi Teratake[1], Chisa Ozeki[1], Yuta Hasegawa[1], Yoshiharu Sato[2], Masayasu Kitahashi[2], Lisa Fujimura[3], Haruko Watanabe-Takano[3], Akemi Sakamoto[1,3], Masafumi Arima[2], Takeshi Tokuhisa[2], and Masahiko Hatano [1, 3,*]

[1]Department of Biomedical Science, [2]Developmental Genetics, Graduate School of Medicine, Chiba University,

[3]Biomedical Research Center, Chiba University

***To whom correspondence should be addressed: Department of Biomedical Science, Graduate School of Medicine, Chiba University, 1-8-1 Inohana, Chuo-ku, Chiba city, Chiba 260-8670, Japan. Tel.: 81-43-226-2950; FAX: 81-43-226-2953; E-mail: hatanom@faculty.chiba-u.jp**

Running title: Nczf targeted disruption in mice

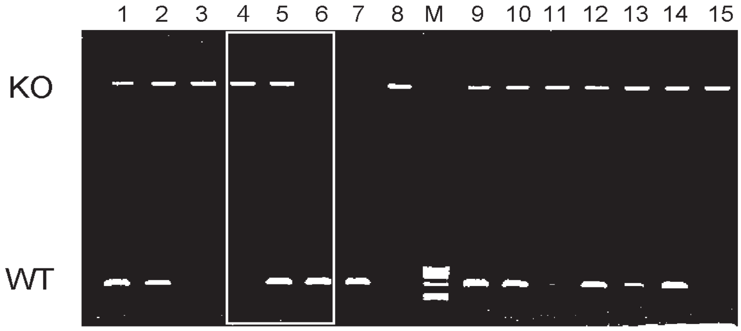

Figure S1
Whole image of gel electrophoresis genotyping Nczf knockout embryos.
Genomic DNA was isolated from E8.5 embryos and PCR was performed with primers specific for wild type and KO allele, respectively. Upper lanes indicate Nczf KO allele (800bp) and lower lanes indicate wild type allele (400bp).
Lanes 4, 5, and 6 were cropped and shown as a representative figure in the main text.
1~15: E8.5 embryos obtained from Nczf heterozygous intercrosses. M: DNA size marker.

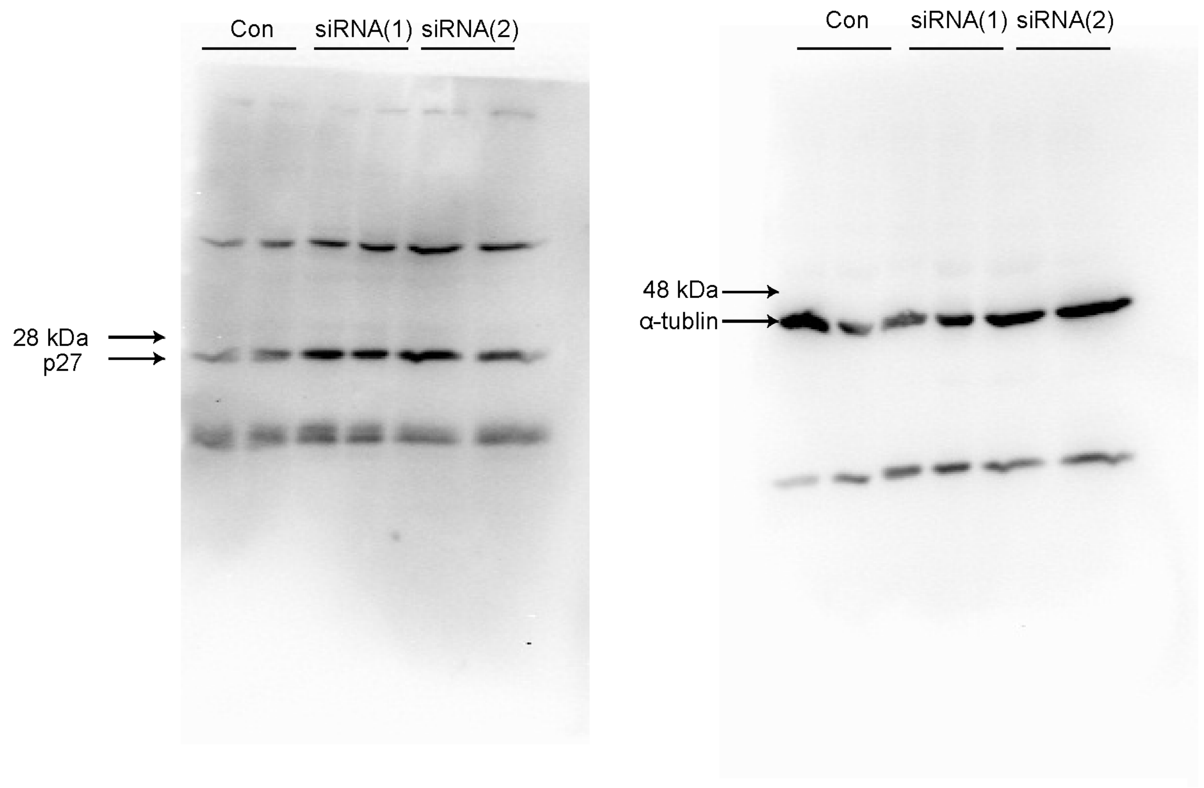

Figure S2
Whole image of western blotting. MEFs from two independent experiments in each siRNA transfection were examined by Western blot analysis. Left figure indicates p27 expression. Right figure shows tubulin expression as a loading control.
